# Evaluation of muscle strength and renal function in survivors of severe COVID-19: A 12-month follow-up study

**DOI:** 10.62838/jccm-2026-0004

**Published:** 2026-04-30

**Authors:** Roberto Marco, Sayane Marlla Silva Leite Montenegro, Daniela Cassula, Tayline Gabriel, Alessandra Lima da Silva Martins, Rosani Teresa de Siqueira e Silva, Rosilene Motta Elias, Maria Aparecida Dalboni

**Affiliations:** Universidade Nove de Julho - Campus Vergueiro, São Paulo, Brazil; Instituto Federal de Pernambuco: Instituto Federal de Educacao Ciencia e Tecnologia de Pernambuco

**Keywords:** post-COVID-19, severe illness, renal function, sarcopenia, quality of life

## Abstract

**Introduction:**

Severe COVID-19 is known to cause kidney injury via ACE2-mediated mechanisms, inflammation, and microvascular damage potentially leading to long-term renal impairment. Critically ill patients are particularly vulnerable to muscle loss and sarcopenia due to immobility, poor nutrition, and cytokine storm–induced catabolism. Post-COVID-19 syndrome often includes fatigue, muscle weakness, and reduced quality of life, yet evidence on long-term outcomes remains limited. This study evaluated kidney function, sarcopenia risk, and quality of life 12 months after intensive care unit (ICU) discharge in patients without pre-existing chronic kidney disease (CKD).

**Methods:**

This retrospective observational cohort included 82 patients without CKD admitted to the ICU between February 2020 and April 2022 who recovered from severe COVID-19. Data collected included serum creatinine, estimated glomerular filtration rate (eGFR), and sarcopenia risk assessed via the SARC-CalF (SARC-F combined with calf circumference). Functional outcomes were assessed by SF-36, pain by a Visual Analog Scale (VAS), and lower limb strength by the 30-second sit-to-stand test.

**Results:**

The mean age was 52 ± 12 years; 90% were male, 46% had hypertension, and 31% diabetes. At 12 months, patients showed low functional scores (SF-36: 47 ± 21), high pain prevalence (VAS: 57%), reduced lower limb strength (sit-to-stand: 8 ± 5 repetitions), and high sarcopenia risk (SARC-F: 46%). Higher sarcopenia scores correlated with poorer physical functioning (r = −0.60; p < 0.001) and greater pain (r = −0.44; p < 0.001). In 49 patients without hypertension, diabetes, or prior acute kidney injury (AKI), creatinine significantly increased (0.95 ± 0.2 to 1.10 ± 0.2 mg/dL; p = 0.007) and eGFR significantly declined (87 ± 22 to 77 ± 18 mL/min; p = 0.001), representing a mean reduction of 10 mL/min.

**Conclusion:**

Critically ill COVID-19 survivors experienced significant declines in kidney function, muscle strength, and functional capacity, alongside increased pain 12 months post-ICU discharge. These results underscore the need for multidisciplinary follow-up, incorporating nephrology, physiotherapy, and nutritional support.

## Introduction

The COVID-19 pandemic triggered a global health crisis, resulting in millions of deaths and placing unprecedented strain on healthcare systems. Approximately 14% of those infected developed severe symptoms requiring hospitalization, with 5% progressing to a critical condition requiring intensive care and respiratory support. In such instances, the mortality rate reached approximately 61.5% [1,2,3,4,5].

The pathophysiology of COVID-19 is characterized by the presence of the ACE2 receptor in various organs, serving as the main entry mechanism for SARS-CoV-2 [2, 6,7]. In the kidneys, ACE2 is highly expressed in podocytes. Consequently, infection is associated with dysregulation of the renin-angiotensin-aldosterone system (RAAS), a high proinflammatory cytokine release, and microvascular thrombosis, collectively resulting in kidney dysfunction [11,12]. Notably, 15% of hospitalized patients exhibited some kidney function impairment, and one in six required dialysis within 60 days of ICU admission [8].

Furthermore, patients with severe COVID-19 often present with depletion of skeletal muscle mass and strength. The disease is a risk factor for sarcopenia due to decreased physical activity, prolonged bed rest, and the “cytokine storm” (e.g., IL-6, TNF-α), which induces a catabolic state. It is estimated that up to 70% of survivors experience muscular, cognitive, or psychological dysfunction within 12 months after discharge [6,8,12].

### Kidney Dysfunction and COVID-19

The monitoring of biomarkers such as serum creatinine, eGFR), and albuminuria is essential for the early detection of kidney dysfunction [11]. Cohort studies have shown that patients recovering from COVID-19 have a higher risk of developing persistent proteinuria six months after infection (post-COVID-19), even in the absence of a prior history of acute kidney injury (AKI) or chronic kidney disease (CKD) [8,12]. This suggests that COVID-19 may cause glomerular injury or predispose individuals to more rapid CKD progression [13]. SARS-CoV-2 infection impacts multiple organs and systems, particularly emphasizing the interaction between the lungs, myocardium, and kidneys. These systems are interlinked through the cytokine storm and microvascular events, which may culminate in AKI [14]. This multifactorial relationship is a key component in the context of COVID-19 and its long-term complications, such as “long COVID”/postCOVID-19 syndrome [15]. The interaction between cardiovascular and kidney systems in COVID-19 suggests that AKI observed in many patients was not the result of a single pathological pathway, but rather a complex interplay of hypoxia, systemic inflammation, and microvascular damage [16]. However, it remains unclear whether kidney dysfunction in COVID-19 patients can persist beyond the acute phase and become a long-term risk factor for progressive loss of kidney function.

### Musculoskeletal Dysfunction and COVID-19

In patients with COVID-19, the exacerbated inflammatory response contributed to protein catabolism and the lower limb and mass skeletal muscle. A multicenter study reported those patients recovering from COVID-19 had a higher rate of musculoskeletal dysfunction compared to critically ill patients with other etiologies [18]. Sarcopenia, defined as the loss of skeletal muscle mass and function; was observed in 43% of patients evaluated six months after hospital discharge, indicating that COVID-19 represents a risk factor for the development of sarcopenia [19, 20].

### Quality of Life and Post-COVID-19 Recovery

In a cohort study involving patients who had recovered from COVID-19 following hospitalization, 63% of participants reported persistent fatigue or muscle weakness six months after hospital discharge [21]. The main symptoms reported by patients with post-COVID-19 syndrome included chronic fatigue, muscle weakness, particularly in the lower limbs and exercise intolerance [20, 21].

The muscle pain, difficulty performing daily physical activities requiring strength or physical endurance, and changes in respiratory capacity, with frequent reports of dyspnea have been observed in these patients. The combination of these symptoms contributed to a significant decline in quality of life and functional capacity in affected individuals [10].

Despite the description of acute complications, the long-term consequences of infection especially regarding kidney function and sarcopenia 12 months post-discharge remain under-investigated. Thus, this study aimed to evaluate kidney function, sarcopenia, and quality of life in patients without pre-existing CKD who recovered from severe COVID-19.

## Methods

### Study Design and Population

This is a retrospective, observational cohort study conducted at the Military Police Medical Center of São Paulo, Brazil. The use of a convenience sample is acknowledged as a limitation. Inclusion criteria were: patients ≥ 18 years old, with severe COVID-19 (WHO guidelines) confirmed by RT-PCR between February 2020 and April 2022, who recovered after ICU admission. Non-inclusion criteria were: pre-existing kidney disease, chemotherapy, pregnancy, recent surgery, previous oxygen use, or musculoskeletal disabilities.

### Data collection

**Fig. 1.**
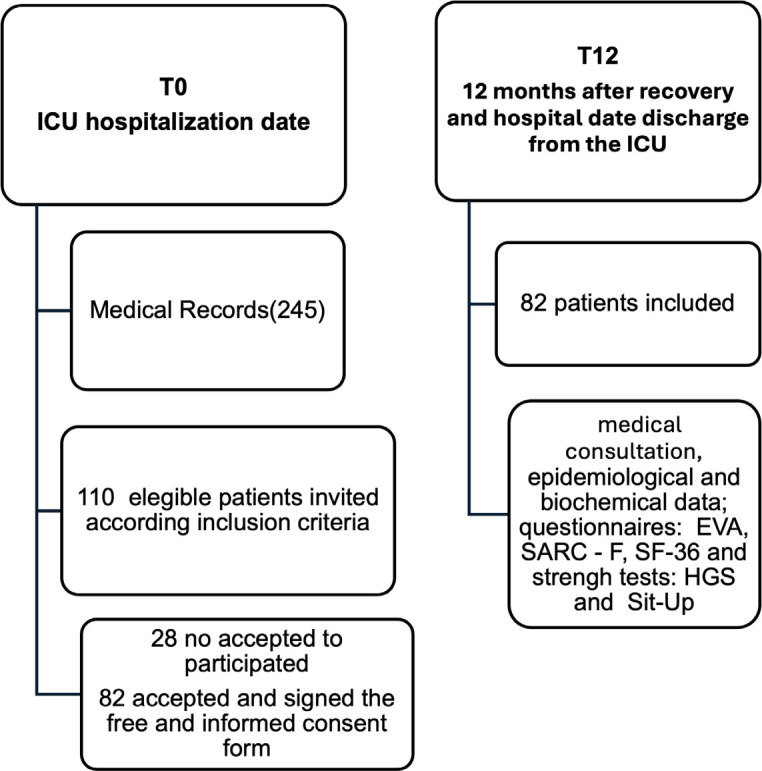
Study design At T0 (ICU admission), 245 medical records were screened, resulting in 110 eligible participants, of which 28 no accepted to participate and 82 were included with complete data. At T12 (12 months after ICU discharge)

T0 data (ICU admission) were extracted from medical records. T12 data (12 months post-discharge) were collected during a scheduled medical consultation. Of 110 eligible patients, 28 declined to participate, and 82 were included (Figure 1).

### Assessments

Renal Function: Serum creatinine was measured, and eGFR was calculated using the CKD-EPI equation.

Sarcopenia (SARC-CalF): Sarcopenia risk was assessed using the SARC-F questionnaire combined with calf circumference (CC). To enhance sensitivity, specific cut-offs were applied: +10 points if CC < 33 cm (women) or < 34 cm (men). A total score ≥ 4 indicated sarcopenia risk.

Muscle Strength: Handgrip strength (HGS) was measured using a digital dynamometer (Saehan). Impairment was defined as < 16 kgf for women and < 27 kgf for men. The 30-second sit-to-stand test was used for lower limb strength, with < 15 repetitions considered abnormal.

Quality of Life: Assessed via SF-36 (functional capacity, physical aspects, and pain domains), VAS for pain, and the Duke Activity Status Index (DASI).

### Duke Activity Status Index (DASI) questionnaire

The DASI questionnaire contains 12 questions about the functionality of the individual’s daily and common activities. This questionnaire was developed in a sample of American adults who self-reported their daily activities and has a score that ranges from 0 to 58.2, where the higher the score, the greater the individual’s functionality [26].

### Visual Analogue Scale (VAS) for pain

The Visual Analogue Scale (VAS) for pain establishes a numerical value in relation to how much pain the individual feels, and the patient can demonstrate their feelings of pain through images (the images show a scale from zero to ten) where the higher the score, the greater the pain.

### Short Form 36 Questionnaire (SF-36)

The SF-36 questionnaire is an instrument that assesses quality of life through 36 items and 8 subscales, encompassing functional capacity, physical aspects, pain, general health status, vitality, social aspects, emotional aspects, relationship between health and work, activities of daily living, mental health and another comparative assessment question between current health conditions and those from up to one year ago, and has been used to assess patients with COVID-19. The score ranges from: 0 = worst and up to a maximum of 100 = best; for each domain (Raw scale) [27,28]. According to the objective of the present study, only functional capacity, physical aspects and pain were analyzed.

### Simple Questionnaire to Rapidly Diagnose Sarcopenia (SARC-F)

The SARC-F questionnaire is widely used to measure the risk of sarcopenia, where five components are evaluated: strength, walking assistance, getting up from a chair, climbing stairs and falling in the last 6 months. Each type of item can score 0, 1 and 2 according to the degree of difficulty or frequency. In the present study, the SARC-F score was calculated based on the calf circumference measurements. Specific cut-off points were applied to enhance diagnostic sensitivity: women received 10 points if the calf measurement was less than 33 cm, while men received 10 points if the measurement was less than 34 cm. These points were integrated into the standard SARC-F scoring system. If the calf measurement was equal to or greater than 33 cm for women and 34 cm for men, the score assigned was zero. The total SARC-F score ranges from 0 to 20. Individuals are classified into different levels of risk for sarcopenia, with those with a score less than or equal to 3 having low risk, those with a score between 4 and 7 having some risk, and those with a score equal to or greater than 8 having high risk [29,30]. For this study, we considered a score equal to or greater than 4 to indicate the risk of sarcopenia.

### Hand Grip Strengh (HGS)

Peripheral muscle strength was assessed using hand grip strength (HGS) using digital dynamometry (Saehan dynamometer, São Paulo, Brazil), in accordance with the recommendations of the American Society of Hand Therapists. Participants were properly instructed and seated without support with their knees flexed at 90°, in an upright posture and with their elbow flexed at 90° close to the trunk. To perform the test, individuals were asked to use the strength of their dominant and non-dominant hands to apply force by pressing the device, generating a 5-second plateau. The process was then repeated 3 times for the dominant and non-dominant hands to obtain the most acceptable and reproducible measurement. The abnormal assessment standard for palmar grip strength is <16 kgf for women and <27 kgf for men, with the strength record established in kilograms/force [kg/f] [29].

### The 30-second sit-to-stand test

The 30-second sit-to-stand test allow for a short period of time to assess flexibility of the lower limb joints, lower limb strength, balance, motor coordination, and the relationship between muscle power and body weight – at the same time, characterizing minimum functional muscle fitness. The test was performed for 30 seconds (short Sit and up test) using a chair with a standard height of 46cm – 48cm positioned against a wall, where the participant was asked to perform sit and stand movements [31]. The knees and hips should be flexed at 90 degrees, and the feet should be flat on the floor and the participant’s hands should rest on their hips, and no support should be used. Over the course of 30 seconds, the participant sat and stood up from the chair repeatedly, as quickly as possible. The test began after a verbal command, and the number of repetitions was counted and recorded. ≥15 repetitions in 30 seconds are considered a normal evaluation standard [32].

### Statistical analysis

The normality of the variables was verified using the Kolmogorov-Smirnov test. Continuous data were presented as mean and standard deviation or median and percentiles 25.75. Categorical data were presented as absolute value and percentage. The comparison of continuous variables over time at T0 and T12 was performed by paired T-test (or Wilcoxon) and categorical data by McNemar’s test. The correlation between the independent variables was performed by Pearson or Spearman according to the data distribution. Values of p < 0.05 were considered statistically significant. The analysis was carried out using SPSS version 28.0 software.

## Results

Demographic data (Table 1) showed a predominantly white (87%), male (90%) population with obesity (56%). Importantly, 93% of patients had received at least two vaccine doses. During the ICU stay, 20% developed AKI and 10% required hemodialysis

**Table 1.** Epidemiological and clinical data of the severely ill individuals eligible for the study at the time of infection (T0) (n = 82)

**Variables**	**n = 82**
Age (years)	52 ±12
Gender (male)	75 (90%)
Race (white)	72 (87%)
BMI (Kg/m^2^)	31 (22–55)
≥18,5 ate 24,9 Kg/m^2^	9 (11%)
≥25 ate 29,9 kg/m	27 (33%)
≥30 kg/m^2^	46 (56%)
Hospitalization in the ICU (days)	9 ± 8
Hypertension (%)	38 (46%)
Diabetes Mellitus (%)	26 (31%)
Cardiovascular disease (%)	5 (6%)
Ventilatory support (%)	15 (18%)
Drug Vasoactive use (%)	20 (24%)
Vacination (Doses) (%)	6 (7%)
0–1 doses	76 (93%)
≥ 2 doses	
Acute kidney disease (%)	16 (20%)
Hemodialysis	8 (10%)

After 12 months, a significant increase in sodium, potassium, albumin and a decrease in blood glucose were observed. However, these changes represented the reestablishment of the parameters to the levels observed in the previous phase. We observed a trend of declining GFR at T12 compared to T0 (p = 0.07) and that approximately 15% of patients had GFR ≤ 59 ml/min at both T0 and T12 (Table 2).

**Table 2.** Biochemical data of the survivors’ patients with severe COVID-19 at T0 (date of ICU admission) and 12 months (T12) after ICU discharge

**Variables**	**T0**	**T12**	**p**
Creatinine (mg/dL)	1.10 ± 0.47	1.12 ± 0.24	0.67
Urea (mg/dL)	39 ± 18	41 ± 12	0.31
Sodium (Na) (mmol/L)	137 ± 3	141 ± 3	< 0.001
Potassium (K) (mmol/L)	3.9 ± 0.6	4.4 ± 0.4	< 0.001
Glucose (mg/dL)	124 ± 36	117 ± 42	< 0.001
Albumin (g/dL)	2.5 ± 0.6	4.5 ± 0.3	< 0.001
eGFR (mL/min) (n/%)	83 ± 23	78 ± 18	0.07
eGFR ≤ 59 (mL/min) (n/%)	12/14%	13/16%	0.98

eGFR = estimated glomerular filtration rate

**Fig.2.**
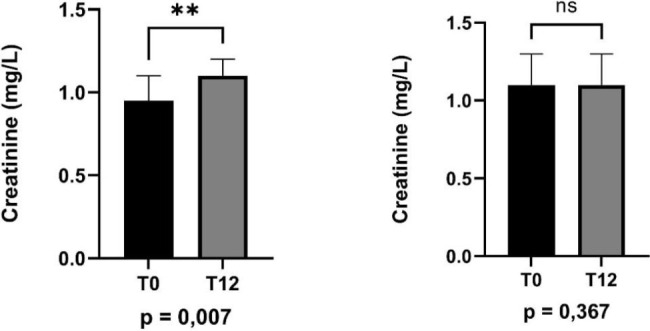
Variation in serum creatinine (mg/dL) in patients with and without comorbidities (hypertension, diabetes, and AKI) between T0 and T12.

At T12, there was a trend toward declining eGFR (p = 0.07) in the total cohort. However, in the subgroup of 49 patients without comorbidities (hypertension, diabetes) or prior AKI (Table 3), a significant reduction in eGFR was observed (87 ± 22 to 77 ± 18 mL/min; p = 0.001) and an increase in serum creatinine (Figure 2).

**Table 3.** Laboratory parameters at T0 and T12 excluding patients with diabetes, systemic arterial hypertension, chronic kidney disease and acute renal failure (n = 49)

**Variables**	**T0**	**T12**	**p**
Creatinine (mg/dL)	0.95 ± 0.2	1.10 ± 0.2	0.007
Urea (mg/dL)	37 ± 15	40 ± 13	0.343
Sodium (Na) (mmol/L)	137 ± 3	141 ± 3	< 0.001
Potassium (k) (mmol/L)	4.00 ± 0.6	4.36 ± 0.4	0.032
Glucose (mg/dL)	113 ± 20	103 ± 14	0.007
Albumin (g/dL)	2.6 ± 0.6	4.6 ± 0.4	< 0.001
eGFR (mL/min) (n/%)	87 ± 22	77 ± 18	0.001

eGFR = estimated glomerular filtration rate

Regarding functional outcomes (Table 4), 57% of patients reported significant pain (VAS ≥ 5). Furthermore, 46% of the survivors were identified as having a high risk of sarcopenia based on SARC-CalF scores. Lower limb strength was notably reduced, with an average of only 8 ± 5 repetitions in the sit-to-stand test. Specifically, 82% of patients performed below the age-matched normal standard for HGS.

**Table 4.** SF36 quality of life assessment (SF36 - functional capacity and pain), pain scale (EVA), functional capacity (DASI), muscle strength - dominant handgrip strength (FPP D) and 30-second sit-to-stand test and risk of sarcopenia (SARC - F), 12 months after the date of recovery from COVID 19 (n = 82)

**Variables**	**T12**
Quality of live	
SF36 – Funcional Capacity	47 ± 21
SF36 – Pain	47 ± 18
Pain Scale	
Visual Analog Scale - 5 (n/%)	47 / 57%
Muscle Strenght	
Handgrip Strenght (kgf)	18 ± 6
The 30-second sit-to-stand test	8 ± 5
Functional Capacity	
Duke Activity Status Index (score)	44 ± 11
Risk of Sarcopenia	
SARC – F (escore ≥ 4)(n/%)	37 / 46%

**Fig. 3.**
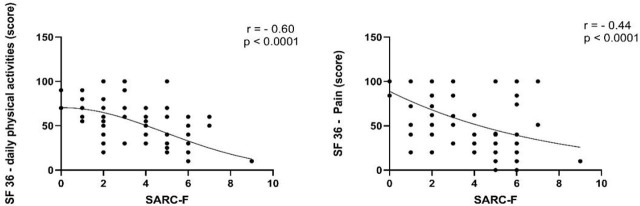
Correlation between sarcopenia risk score (SARC-F) and low Daily Functional capacity score (SF36) (T12) and correlation between score sarcopenia risk score (SARC-F) and pain score (SF36) (T12).

Regarding the correlations, the coefficients (r = −0.60 for physical functioning and r = −0.44 for pain) indicate strong clinical associations. These values, paired with the reported p-values, suggest that the risk of sarcopenia is a robust predictor of poor functional outcomes in this cohort (Figure 3).

## Discussion

The aim of this study was to assess renal function, sarcopenia, and quality of life in survivors of severe COVID-19 after 12 months post-ICU discharge. As expected, we observed that after 12 months of recovery from COVID-19 infection, sodium, potassium, and albumin levels returned to their normal concentrations. In the overall population studied, there was a trend toward decreased GFR after 12 months (p = 0.07). However, when excluding patients with diabetes, hypertension, and those who developed AKI during hospitalization, we observed a significant decline in eGFR (p < 0.001) and an increase in creatinine (p < 0.001) after 12 months of recovery and ICU discharge. Kidney failure has been reported as one of the common complications in patients with severe COVID-19 during hospitalization [14]. However, few studies have evaluated the long-term impact of COVID-19 on kidney function. In this context, two studies reported increased proteinuria and albuminuria in patients who recovered from COVID-19 infection 6 months later [7,8].

Some studies suggest that microthrombotic and inflammatory mechanisms during acute infection may be involved in post-COVID-19 kidney injury [17,34]. Furthermore, experimental studies and histopathological analyses have reported that activation of the renin-angiotensin-aldosterone system (RAAS) by SARSCoV-2 may also contribute to tubular injury and interstitial renal fibrosis in postCOVID-19 patients [17,35]. In this study, we did not evaluate the mechanisms that could be involved in renal injury caused by COVID-19 after 12 months, such as activation of the inflammatory response, coagulation cascade, or analysis of proteinuria. However, we observed a decline in eGFR after 12 months from ICU discharge due to COVID-19. We can suggest that the entry of the SARS-CoV-2 virus through its receptor (ACE2) expressed in podocytes during acute infection may have compromised a population of glomeruli, reflecting lower renal filtration in these patients. We observed a decline of approximately 10 mL/min in GFR after 12 months of recovery from acute COVID-19 infection, which corresponds to an average 10% decrease in renal filtration. Although we observed these changes, it is important to note that creatinine and eGFR were still within the normal range according to sex and age in this population. However, CKD has a slow progression, and the long-term impact of COVID-19 on kidney function is still not fully understood. Therefore, based on our results, we suggest that the monitoring of kidney function biomarkers should be advised in patients who had severe COVID-19 to evaluate whether SARS-CoV-2 could be a risk factor for altering kidney function, in addition to the known risk factors such as age, diabetes, and hypertension [11].

COVID-19 has not only resulted in a significant mortality rate, especially among critically ill patients, but long-term sequelae that affect the quality of life and functional capacity of survivors have also been reported [34]. Studies have shown that COVID-19 infection can lead to multisystem complications, including sarcopenia, which are particularly relevant to the quality of life of these patients [35]. Sarcopenia has emerged as a significant concern among COVID-19 survivors. It is estimated that up to 43% of patients develop sarcopenia after hospital discharge, which can compromise their functional capacity and quality of life [11]. In fact, in our study, after 12 months of COVID-19 infection, 57% of patients reported pain, 50% had a score ≥ 5 for sarcopenia risk (SARC-F) and decreased lower limb strength. Furthermore, we observed negative correlations between high sarcopenia risk score (SARC-F) between low daily functional capacity score and pain score; suggesting that the increased risk of sarcopenia, could reflect a decreased skeletal muscle mass, and impact the negatively the daily functional capacity functions and increased levels of pain. Similarly, other studies have reported persistent fatigue, functional limitations, and pain [36] even one year after COVID-19 infection, as assessed by instruments/questionnaires VAS and SF-36, respectively [14,15,16]. A metaanalysis study in critically ill patients post-COVID-19 also reported a significant reduction in muscle strength, with direct impacts on mobility and return to daily activities [37]. Recent studies demonstrate that the exacerbated inflammatory response with activation of inflammatory cytokines, such as IL-6 and TNF-α, during infection [12,13], associated with mitochondrial dysfunction and increased protein catabolism, can be perpetuated post-infection [38] and be one of the factors that delay the recovery of skeletal muscle mass and strength in this population [39,40]. Thus, the loss of skeletal muscle mass and strength may be associated with a worse quality of life of patients recovered from severe COVID-19 infection [41,42]. Indeed, our observation of a higher risk of sarcopenia and loss of skeletal muscle strength after 12 months of recovery from the infection suggests that COVID-19 may contribute to a worse quality of life in this population. The high risk of long-term sarcopenia observed in this population requires early and intensive muscle rehabilitation interventions, including functional physical therapy and nutritional support, involving the preservation of lean mass and recovery of muscle strength. The results of this study reinforce the repercussions of severe COVID-19 in surviving patients, revealing the persistence of multisystem dysfunctions that challenge clinical practice and long-term therapeutic management. The alteration in kidney function, evidenced by the significant increase in creatinine levels and decrease in eGFR after 12 months, suggests that COVID-19 infection may be a risk factor for CKD in a significant proportion of patients. This data highlights the importance of continuous nephrological surveillance, with periodic assessments of creatinine and eGFR and the adjustment of external therapies to preserve kidney function, preventing progression to more advanced improvements in kidney failure.

Study limitations: a) the sample size, although adequate to detect statistical trends, may not reflect the full heterogeneity of post-COVID patients; b) This study was retrospective and observational and did not evaluate instruments for sarcopenia, muscle strength and quality of life at the time of infection in the ICU; c) We did not investigate proteinuria before and after 12 months after COVID19; d) we did not evaluate inflammatory biomarkers to observe association with the sequelae studied.

Our findings demonstrate that 12 months after ICU discharge, survivors of severe COVID-19 exhibit significant persistent functional and renal impairments. These results align with major long-COVID cohorts, such as the UK PHOSP-COVID study and Huang et al. (Lancet 2021), which highlighted that a substantial proportion of survivors do not fully recover their baseline health status even one year later [41].

The observed decline in eGFR, particularly in previously healthy individuals, suggests that COVID-19 may accelerate renal decline in some survivors. This is likely driven by the persistent dysregulation of the RAAS and chronic low-grade inflammation following the initial cytokine storm.

Muscle weakness was pervasive. The high risk of sarcopenia (46%) and low sit-to-stand performance (8 reps) reflect the profound impact of critical illness catabolism. The strong correlation between sarcopenia and physical functioning (r = −0.60) underscores the clinical relevance of early screening for musculoskeletal dysfunction.

In summary, this study emphasizes the importance of a longitudinal approach in the management of critically ill patients after COVID-19, considering not only the assessment of kidney function and skeletal muscle mass (sarcopenia), but also functional capacity and pain, which have implications for quality of life, but has limitations that must be acknowledged. First, the population was highly homogenous (predominantly males, mean age 52, which limits the generalizability of the findings to broader populations. Second, the single-center design in São Paulo may reflect local clinical practices. Most importantly, we lacked baseline pre-COVID-19 data for renal function and muscle strength, preventing a direct comparison with the patients’ true physiological state before infection. Finally, the use of convenience sampling may introduce selection bias.

## Conclusion

Severe COVID-19 survivors face a high burden of renal decline, muscle weakness, and chronic pain 12 months after ICU discharge. Clinicians should prioritize long-term multidisciplinary monitoring to mitigate the progression of these complications.

## References

[1] Centers for Disease Control and Prevention (2025). People with Certain Medical Conditions and COVID-19 Risk Factors [Internet].

[2] Jordan RE, Adab P, Cheng KK (2020). Covid-19: risk factors for severe disease and death. BMJ.

[3] Raschke RA (2021). Discriminant accuracy of the SOFA score for determining the probable mortality of patients with COVID-19 pneumonia requiring mechanical ventilation. JAMA.

[4] Brazil. Ministry of Health (2025). Coronavirus Panel: COVID-19 data in Brazil [Internet].

[5] Grasselli G (2020). Baseline characteristics and outcomes of 1591 patients infected with SARS-CoV-2 admitted to ICUs of the Lombardy region, Italy. JAMA.

[6] Gabarre P (2020). Acute kidney injury in critically ill patients with COVID-19. Intensive Care Med.

[7] Chakraborty I, Maity P (2020). COVID-19 outbreak: migration, effects on society, global environment and prevention. Sci Total Environ.

[8] Veras FP (2022). Neutrophil extracellular traps (NETs): importance in pathogenesis and potential therapeutic target during COVID-19 [thesis].

[9] Xia H, Lazartigues E (2010). Angiotensin-converting enzyme 2: central regulator for cardiovascular function. Curr Hypertens Rep.

[10] Nalbandian A (2021). Post-acute COVID-19 syndrome. Nat Med.

[11] Bowe B (2021). Kidney outcomes in long COVID. J Am Soc Nephrol.

[12] Yang D (2020). Physical function in kidney transplantation: current knowledge and future directions. Curr Transplant Rep.

[13] Gutiérrez-Chamorro L (2021). SARS-CoV-2 infection modulates ACE2 function and subsequent inflammatory responses in swabs and plasma of COVID-19 patients. Viruses.

[14] Hirsch JS (2020). Acute kidney injury in patients hospitalized with COVID-19. Kidney Int.

[15] Andrade RO (2020). The effects of Covid-19: months after overcoming the acute phase of the disease, some patients still have persistent complications in the lungs, heart or brain. Rev Pesquisa FAPESP [Internet].

[16] Ronco C, Reis T, Husain-Syed F (2020). Management of acute kidney injury in patients with COVID-19. Lancet Respir Med.

[17] Su H (2020). Renal histopathological analysis of 26 postmortem findings of patients with COVID-19 in China. Kidney Int.

[18] McWilliams D (2020). Rehabilitation levels in COVID-19 patients admitted to intensive care requiring invasive ventilation: an observational study. Ann Am Thorac Soc.

[19] Nelke C (2019). Skeletal muscle as potential central link between sarcopenia and immune senescence. EBioMedicine.

[20] Ramírez-Vélez R (2023). Reduced muscle strength in patients with long COVID-19 syndrome is mediated by limb muscle mass. J Appl Physiol.

[21] Pecly IMD (2021). A review of COVID-19 and acute kidney injury: from pathophysiology to clinical results. J Bras Nefrol.

[22] Evans RA (2021). Physical, cognitive, and mental health impacts of COVID-19 after hospitalisation (PHOSP-COVID): a UK multicentre, prospective cohort study. Lancet Respir Med.

[23] World Health Organization (2020). Laboratory testing for 2019 novel coronavirus (2019-nCoV) in suspected human cases [Internet].

[24] World Health Organization (2020). Laboratory testing for coronavirus disease 2019 (COVID-19) in suspected human cases: interim guidance, 2 March 2020 [Internet].

[25] Levey AS (2009). A new equation to estimate glomerular filtration rate. Ann Intern Med.

[26] Hlatky MA (1989). A brief self-administered questionnaire to determine functional capacity (the Duke Activity Status Index). Am J Cardiol.

[27] Scott J, Huskisson EC (1976). Graphic representation of pain. Pain.

[28] Aquino ALN, Silva ROE (2021). Evaluation of the quality of life of coffee growers in Poço Fundo-MG with the SF-36 instrument during the COVID-19 pandemic. Research, Society and Development.

[29] Malmstrom TK, Morley JE (2013). SARC-F: a simple questionnaire to rapidly diagnose sarcopenia. J Am Med Dir Assoc.

[30] Kirk B (2024). An executive summary on the global conceptual definition of sarcopenia.

[31] Pagotto V (2018). Calf circumference: clinical validation for evaluation of muscle mass in the elderly. Rev Bras Enferm.

[32] Piquione FS (2021). Evaluation of peripheral muscle strength of patients hospitalized with respiratory diseases submitted to early mobilization. Research, Society and Development.

[33] Marincolo JCS (2011). Indicators of frailty and time spent in activities in the elderly: data from FIBRA Campinas [Master’s Thesis].

[34] Lombardi R (2014). Acute kidney injury in Latin America: a view on renal replacement therapy resources. Nephrol Dial Transplant.

[35] Hirsch JS (2020). Acute kidney injury in patients hospitalized with COVID-19. Kidney Int.

[36] Smith BH, Belton JL (2024). Patient engagement in pain research: no gain without the people in pain. Pain.

[37] Evans DW (2025). Where is the pain? Spatial patterns of pain co-occurrence in a population-based study of 4833 pain drawings incorporating network analysis. Pain.

[38] Nydahl P (2017). Safety of patient mobilization and rehabilitation in the intensive care unit: systematic review with meta-analysis. Ann Am Thorac Soc.

[39] Yin Y (2024). Health consequences among COVID-19 convalescent patients 30 months post-infection in China. Zoonoses.

[40] Toledano JM (2023). Antioxidant and immune-related implications of minerals in COVID-19: a possibility for disease prevention and management. Antioxidants (Basel).

[41] Huang L (2021). 1-year outcomes in hospital survivors with COVID-19: a longitudinal cohort study. Lancet.

[42] De Blasio F (2021). Sarcopenia in post-acute COVID-19 patients: evidence from a multicenter study. J Clin Med.

